# Undocumented Anxiolytic and Sedative Use and Its Association With Depressive and Anxiety Symptoms in Primary Care Patients Without Registered Mental Disorders

**DOI:** 10.7759/cureus.115493

**Published:** 2026-08-31

**Authors:** Sabina Ademovic Dzambic, Danijel Gajic, Emina Bajric Custo, Branislav Milic, Senada Selmanovic, Dzana Nuhanovic, Lejla Hodzic, Inela Kadric, Sabina Cemalovic, Sanda Kreitmayer Pestic

**Affiliations:** 1 Department of Family Medicine, Public Health and Educational Institution Health Center “Dr. Mustafa Šehović” Tuzla, Tuzla, BIH; 2 Department of Family Medicine, Health Center Orašje, Orašje, BIH; 3 Department of Family Medicine, Health Center Lukavac, Lukavac, BIH; 4 Department of Psychiatry, Health Center Orašje, Orašje, BIH; 5 Faculty of Medicine, University of Tuzla, Tuzla, BIH; 6 Clinic for Radiology and Nuclear Medicine, University Clinical Center Tuzla, Tuzla, BIH; 7 Department of Pediatrics, University Clinical Center Tuzla, Tuzla, BIH; 8 Department of Cardiology, Kantonalna bolnica Dr. Irfan Ljubijankić, Bihać, BIH; 9 Department of Family Medicine, Public Health and Educational Institution Health Center “Dr. Mustafa Šehović”, Tuzla, BIH

**Keywords:** anxiety, anxiolytics, depression, primary healthcare, sedatives

## Abstract

Background

Undocumented and self-initiated use of psychotropic medications represents a growing public health challenge in primary care. This study aimed to evaluate the prevalence of self-reported occasional use of anxiolytics and/or sedatives among primary healthcare (PHC) patients without a previously registered diagnosis of a mental disorder and without a documented prescription for these medications in their medical charts. Additionally, we investigated the associations of undocumented medication use and sociodemographic parameters with the severity of depressive and anxiety symptoms.

Methods

A cross-sectional study was conducted between January 31, 2026, and March 31, 2026, at the Public Health Institution Health Center “Dr. Mustafa Šehović” Tuzla, Bosnia and Herzegovina, enrolling 221 adult PHC patients (N = 221). Depressive and anxiety symptoms were evaluated using the Patient Health Questionnaire-9 (PHQ-9) and Generalized Anxiety Disorder-7 (GAD-7) screening tools. Participants were categorized as users (n = 60) or non-users (n = 161) based on self-reported occasional medication intake. Nonparametric statistical tests (the Mann-Whitney U test and Kruskal-Wallis H test with Bonferroni-adjusted post hoc comparisons) were used for group comparisons.

Results

Occasional undocumented use of anxiolytics and/or sedatives was reported by 27.1% (n = 60) of participants. Medication users had significantly higher scores on both the PHQ-9 (median 10.0 (IQR = 7) vs. 4.0 (IQR = 5); p < 0.001) and GAD-7 (median 8.5 (IQR = 9) vs. 4.0 (IQR = 6); p < 0.001) than non-users. Among medication users, a statistically significant association was identified between educational level and depressive symptom severity (H = 6.515, df = 2, p = 0.038); post hoc analysis confirmed that participants with secondary education had significantly higher PHQ-9 scores than those with higher education (p = 0.036). Sex, age, and marital status showed no significant associations with symptom severity within the user group.

Conclusion

More than a quarter of primary care patients without a documented mental disorder or prescription for anxiolytics and/or sedatives reported occasional use of these medications and had elevated depressive and anxiety symptom scores. These findings emphasize the clinical need for proactive, comprehensive medication history-taking in family medicine to identify undisclosed psychotropic medication use and underlying psychological distress.

## Introduction

Mental disorders, primarily depressive and anxiety disorders, represent a leading global public health burden, with a substantial proportion of affected individuals lacking access to adequate healthcare. Primary healthcare (PHC) plays a crucial role in their early identification, serving as the patient’s initial point of contact with the healthcare system [1,2]. However, depressive and anxiety disorders frequently remain undetected in primary care, particularly when patients present predominantly with somatic complaints [3].

Anxiolytics and sedatives, particularly benzodiazepines, are widely used in the treatment of anxiety and sleep disorders; however, their use requires caution because of the risks of tolerance, dependence, and adverse effects [4,5]. Official data from the Agency for Medicinal Products and Medical Devices of Bosnia and Herzegovina confirm their persistently high outpatient consumption, reflecting the prominent use of this pharmacotherapeutic group in outpatient settings in Bosnia and Herzegovina [6]. Beyond prescribed therapy, non-medical or self-initiated use of these medications is prevalent across Europe [7,8]. Patterns of self-medication vary across countries depending on healthcare system organization and medication accessibility [9,10]. In Bosnia and Herzegovina, general population data indicate that 20.1% of benzodiazepine users obtain these medications from social sources, such as family or friends, a proportion that reaches 41.3% among young adults [11]. This underscores a heavy reliance on informal distribution channels and points to widespread use outside the formal prescribing system.

For the objective evaluation of depressive and anxiety symptoms in PHC, the use of validated instruments is recommended, such as the Patient Health Questionnaire-9 (PHQ-9) for depressive symptoms (score range: 0-27) and the Generalized Anxiety Disorder-7 (GAD-7) for anxiety symptoms (score range: 0-21) [12,13]. Systematic reviews confirm their utility for screening, emphasizing that elevated scores do not constitute a standalone diagnosis but instead indicate the need for further clinical assessment [14-16]. Given that psychological symptoms are intertwined with sociodemographic variables and chronic medical conditions [17-19], there is a clear rationale for investigating the occasional use of anxiolytics and/or sedatives without documented prescription therapy in patients’ medical records.

The aim of this study was to evaluate the prevalence of self-reported, occasional use of anxiolytics and/or sedatives among adult PHC patients without a previously registered diagnosis of a mental disorder and without documented prescribed therapy for medications from this class in their medical records, as well as to analyze the socio-demographic and clinical characteristics of users. Specific objectives were to examine differences in PHQ-9 and GAD-7 scores between users and non-users of anxiolytics and/or sedatives, and to investigate the association of sex, age, marital status, and educational level with PHQ-9 and GAD-7 scores among users of anxiolytics and/or sedatives.

## Materials and methods

Study design and study population

A cross-sectional study was conducted between January 31, 2026 and March 31, 2026, at the Center for Education and Professional Development of the Public Health Institution Health Center “Dr. Mustafa Šehović” Tuzla.

A total of 271 patient medical records were initially reviewed for study eligibility using a consecutive sampling method, whereby all adult patients presenting for routine or non-emergency consultations during the study period were sequentially screened. Based on this preliminary database screening, 50 patients were excluded because of an existing documented diagnosis of a mental disorder. The remaining 221 eligible adult patients (aged 18 years or older) who visited a family medicine physician during the study period met all baseline criteria and were invited to participate. All 221 invited patients provided voluntary informed consent and fully completed the study questionnaires (0% refusal rate and no incomplete questionnaires). The detailed selection process is illustrated in Figure 1.

**Figure 1:**
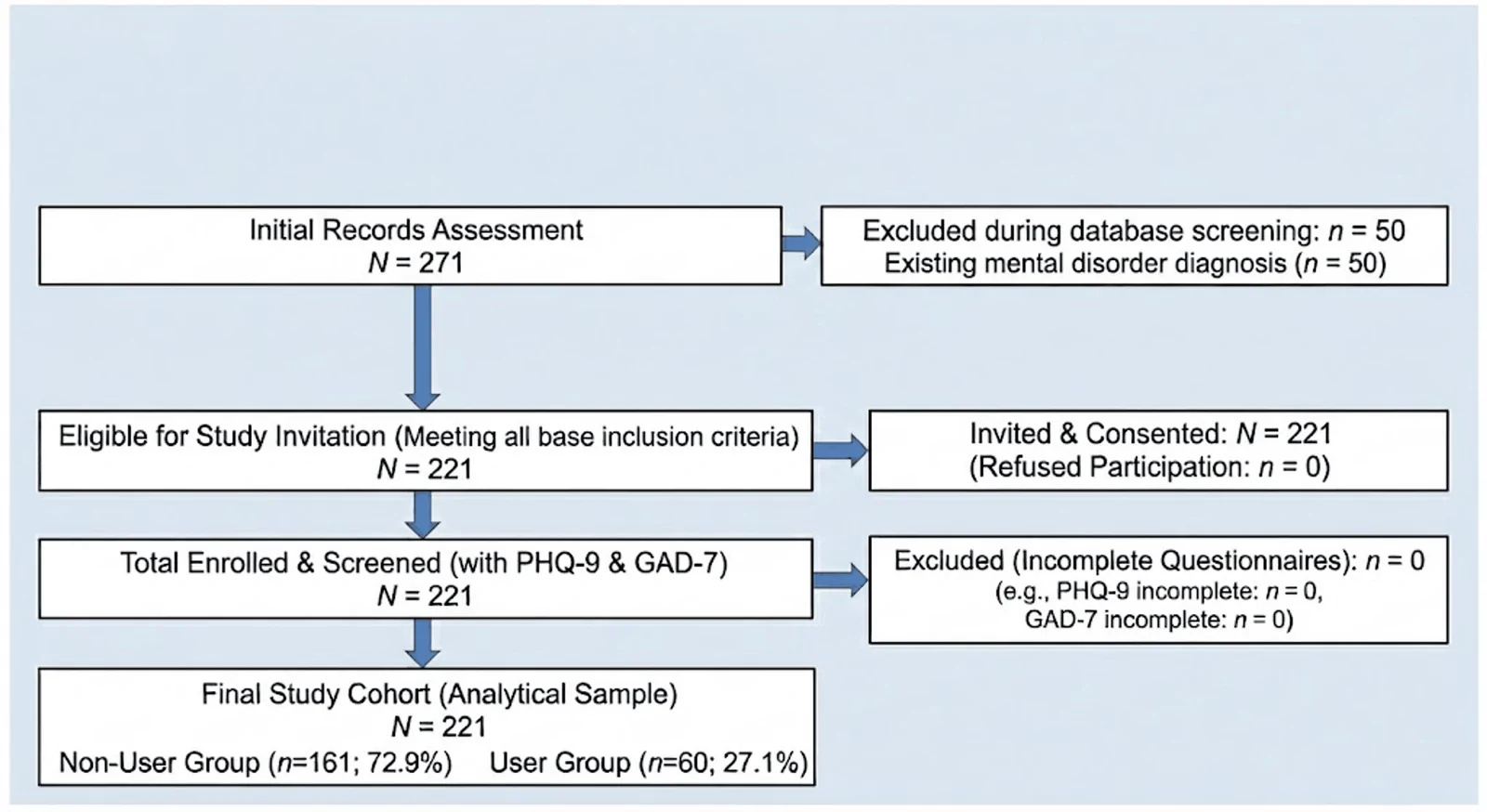
Participant selection flowchart. Flowchart illustrating patient screening, exclusion based on medical-record review, enrollment, and the final study sample distribution (N = 221). PHQ-9: Patient Health Questionnaire-9; GAD-7: Generalized Anxiety Disorder-7.

The inclusion criteria comprised adult patients (aged 18 years or older) who visited a family medicine physician for any clinical reason during the study period, had no previously registered diagnosis of a mental disorder according to the ICD-10 classification, had no documented prescription for anxiolytics and/or sedatives in their medical records, and provided voluntary informed consent to participate. Conversely, the exclusion criteria comprised patients with an existing diagnosis of a mental disorder, patients undergoing active psychiatric treatment or follow-up, and those with an officially documented prescription for pharmacotherapy from the group of anxiolytics and/or sedatives in their medical charts. Additionally, patients who, because of their health condition, were unable to complete the questionnaire independently or who declined to provide informed consent were excluded.

Data on actual medication use were based on participant self-reports and were not additionally verified through pharmacy records or other sources. Data regarding the precise medication name, dose, duration of use, time of last use, or source of procurement were not systematically collected, which should be considered when interpreting the results.

Participants were divided into two groups: users and non-users of anxiolytics and/or sedatives. The user group (n = 60; 27.1%) consisted of participants who responded affirmatively (“Yes”) to the structured questionnaire item: “Do you occasionally use anxiolytics and/or sedatives (e.g., medications for relaxation, anxiety, or sleep) that are not currently prescribed in your medical record?” Occasional use was operationally defined as self-reported episodic or non-daily consumption of these medications within the preceding 12 months, based on participant self-assessment and without a predefined numerical frequency threshold. The non-user group (n = 161; 72.9%) comprised participants who reported no use of these medications during the same period. For brevity and clarity throughout the text, the terms “users” and “non-users” are used.

Data collection and instruments

Data were collected using a structured questionnaire consisting of three parts. The first part addressed the sociodemographic and clinical characteristics of the participants, including sex, age, marital status, and educational level. The second and third parts assessed depressive and anxiety symptoms, respectively.

Assessment of depressive and anxiety symptoms

Depressive symptoms were evaluated using the PHQ-9, originally developed by Kroenke K et al. [12]. The PHQ-9 is a nine-item self-report module designed to assess the nine Diagnostic and Statistical Manual of Mental Disorders, Fourth Edition (DSM-IV) symptom criteria for major depressive disorder using a four-point Likert scale ranging from 0 (“not at all”) to 3 (“nearly every day”), yielding a total score ranging from 0 to 27.

Anxiety symptoms were assessed using the GAD-7 scale, developed by Spitzer RL et al. [13]. The GAD-7 is a seven-item self-report tool scored using a similar four-point Likert scale ranging from 0 (“not at all”) to 3 (“nearly every day”), with total scores ranging from 0 to 21.

Validated local-language (Bosnian/Croatian/Serbian) adaptations of both the PHQ-9 and GAD-7 were administered.

Both instruments are validated, open-access clinical screening tools. The PHQ-9 and GAD-7 were created by Drs. Robert L. Spitzer, Janet W.B. Williams, Kurt Kroenke, and colleagues, with an educational grant from Pfizer Inc. No permission is required to reproduce, translate, display, or distribute these measures.

In this study, the term “undocumented use” refers strictly to the absence of a recorded prescription for anxiolytics and/or sedatives in the participant’s primary care medical chart at the study center. This designation does not necessarily imply non-medical or self-initiated use, as participants may have obtained prescriptions from private providers, specialists, or other healthcare settings outside the study facility.

Statistical analysis

Data analysis was performed using IBM SPSS Statistics software (version 26). Continuous variables were summarized using descriptive statistics, including the mean ± SD, median, and IQR. The normality of the distributions was assessed using the Shapiro-Wilk and Kolmogorov-Smirnov tests. The Mann-Whitney U test was used to compare two independent groups, and the Kruskal-Wallis H test with Bonferroni-adjusted post hoc comparisons was used to compare multiple groups. Statistical significance was set at p < 0.05.

Ethical considerations

The study was conducted in accordance with the fundamental principles of medical ethics, the code of medical deontology, and the Declaration of Helsinki. The study protocol was approved by the Ethics Committee of the Public Health Institution Health Center “Dr. Mustafa Šehović” Tuzla (decision number: 04-4-584-1; dated January 30, 2026). Participation was entirely voluntary, and all participants signed an informed consent form before inclusion. All collected data were processed anonymously and in accordance with the principles of medical confidentiality. As a safety protocol, any participant who endorsed item 9 of the PHQ-9 (thoughts of self-harm or suicide) or demonstrated markedly elevated depression or anxiety scores was immediately offered a clinical consultation with the attending primary care physician and expedited referral to specialized psychiatric services.

## Results

The study included a total of 221 participants (N = 221), of whom 60 (27.1%) reported using anxiolytics and/or sedatives, while the remaining 161 (72.9%) were categorized as non-users.

Participants who used anxiolytics and/or sedatives had significantly higher scores on both the PHQ-9 and GAD-7 than non-users (Table 1). These primary group comparisons reflect unadjusted analyses. The median PHQ-9 score was 10.0 in the user group (IQR = 7), compared with 4.0 in the non-user group (IQR = 5; p < 0.001). The median GAD-7 score was 8.5 in the user group (IQR = 9), compared with 4.0 in the non-user group (IQR = 6; p < 0.001).

**Table 1:** Differences in PHQ-9 and GAD-7 scores between users and non-users of anxiolytics and/or sedatives. Data are presented as mean ± SD and median with IQR. Between-group differences were evaluated using the Mann-Whitney U test. PHQ-9: Patient Health Questionnaire-9; GAD-7: Generalized Anxiety Disorder-7. *Statistically significant at p < 0.05.

	Anxiolytic and sedative medication users			Non-users			
Parameter	N (%)	(min-max) median (IQR)	Mean ± SD	N (%)	(min-max) median (IQR)	Mean ± SD	p-value
PHQ-9	60 (27.1)	0-22; 10.0 (7)	9.73 ± 5.03	161 (72.9)	0-26; 4.0 (5)	4.92 ± 4.23	<0.001*
GAD-7	60 (27.1)	0-21; 8.5 (9)	9.57 ± 5.69	161 (72.9)	0-21; 4.0 (6)	4.71 ± 4.31	<0.001*

Analyses by sex, age, educational level, and marital status are presented as secondary exploratory analyses. Because of the relatively small number of participants in certain categories (e.g., n = 6 in the <30-year age group and n = 8 in the primary-education group among users), these analyses had limited statistical power, and the subgroup estimates may be unstable. Therefore, the results should be interpreted with caution.

Within the group of anxiolytic and/or sedative users, no statistically significant differences were found between men and women in the severity of depressive (U = 210.0, Z = -1.446, p = 0.148) or anxiety symptoms (U = 232.0, Z = -1.038, p = 0.299). In the non-user group, statistically significant sex differences were observed, with women having significantly higher scores than men on both the PHQ-9 (U = 2240.5, Z = -2.997, p = 0.003) and GAD-7 (U = 2098.5, Z = -3.492, p < 0.001) (Table 2).

**Table 2:** Sex differences in PHQ-9 and GAD-7 scores among users and non-users of anxiolytics and/or sedatives. Differences between men and women were evaluated using the Mann-Whitney U test. PHQ-9: Patient Health Questionnaire-9; GAD-7: Generalized Anxiety Disorder-7. *Statistically significant at p < 0.05.

Anxiolytic and/or sedative use	Parameter	Men, N	Men, mean rank	Women, N	Women, mean rank	Mann-Whitney U	Z	p-value
Yes	PHQ-9	12	37	48	28.88	210	-1.446	0.148
	GAD-7		35.17		29.33	232	-1.038	0.299
No	PHQ-9	64	67.51	97	89.9	2240.5	-2.997	0.003*
	GAD-7		65.29		91.37	2098.5	-3.492	<0.001*

In the analysis of age categories among users of anxiolytics and/or sedatives, no statistically significant differences were found in PHQ-9 (H = 7.135, df = 5, p = 0.211) or GAD-7 scores (H = 7.667, df = 5, p = 0.176). Within the non-user group, a statistically significant difference was observed in PHQ-9 scores across age categories (H = 14.495, df = 5, p = 0.013), whereas no statistically significant difference was found in GAD-7 scores (H = 7.586, df = 5, p = 0.181) (Table 3). Post hoc analysis using Bonferroni correction demonstrated that participants under 30 years of age had significantly higher PHQ-9 scores than those aged 61-70 years. No statistically significant differences in PHQ-9 scores were observed among the other age groups after adjustment for multiple comparisons (all adjusted p > 0.05) (Table 4).

**Table 3:** Differences in PHQ-9 and GAD-7 scores by age among users and non-users of anxiolytics and/or sedatives. Differences across age groups were evaluated using the Kruskal-Wallis H test. PHQ-9: Patient Health Questionnaire-9; GAD-7: Generalized Anxiety Disorder-7. *Statistically significant at p<0.05.

Anxiolityc and Sedative medication use	Parameter	<30 years	31-40 years	41-50 years	51-60 years	61-70 years	>71 years	Kruskal-Wallis H	df	p-value
Yes	PHQ-9, N	6	11	11	7	15	10	7.135	5	0.211
	Mean rank	37.42	30.73	37.18	36	26.57	20.8			
	GAD-7, N	6	11	11	7	15	10	7.667	5	0.176
	Mean rank	36.17	35.77	34.77	26.07	31.4	18.35			
No	PHQ-9, N	34	37	31	26	24	9	14.495	5	0.013*
	Mean rank	103.34	75.15	78.21	84.13	58.21	82			
	GAD-7, N	34	37	31	26	24	9	7.586	5	0.181
	Mean rank	92.54	81.62	80.84	87.17	60.5	72.22			

**Table 4:** Post hoc analysis of PHQ-9 scores by age among non-users of anxiolytics and/or sedatives. Post hoc pairwise comparisons were conducted following the Kruskal-Wallis H test, with Bonferroni correction for multiple comparisons. PHQ-9: Patient Health Questionnaire-9. *Statistically significant at p < 0.05.

Pairwise comparison (years)	Test statistic	Standardized test statistic	Unadjusted p-value	Bonferroni-adjusted p-value
61-70 vs. 31-40	-16.94	-1.16	0.246	1
61-70 vs. 41-50	-20	-1.319	0.187	1
61-70 vs. >71	-23.79	-1.127	0.26	1
61-70 vs. 51-60	-25.92	-1.637	0.102	1
61-70 vs. <30	-45.13	-3.064	0.002	0.033*
31-40 vs. 41-50	-3.06	-0.218	0.827	1
31-40 vs. >71	-6.85	-0.342	0.732	1
31-40 vs. 51-60	-8.98	-0.612	0.541	1
31-40 vs. <30	-28.19	-2.1	0.036	0.536
41-50 vs. >71	-3.79	-0.185	0.853	1
41-50 vs. 51-60	-5.92	-0.389	0.697	1
41-50 vs. <30	-25.13	-1.799	0.072	1
>71 vs. 51-60	2.13	0.098	0.922	1
>71 vs. <30	-21.34	-1.042	0.297	1
51-60 vs. <30	-19.21	-1.309	0.191	1

Educational level demonstrated a statistically significant association with the severity of depressive symptoms (PHQ-9) among users of anxiolytics and/or sedatives (H = 6.515, df = 2, p = 0.038), whereas its association with anxiety symptom severity (GAD-7) was not statistically significant (H = 4.333, df = 2, p = 0.115) (Table 5). Post hoc analysis using Bonferroni correction confirmed that participants with a secondary school education had significantly higher PHQ-9 scores than those with higher education (p = 0.036) (Table 6).

**Table 5:** Differences in PHQ-9 and GAD-7 scores by educational level among users of anxiolytics and/or sedatives. Differences across educational levels were evaluated using the Kruskal-Wallis H test. PHQ-9: Patient Health Questionnaire-9; GAD-7: Generalized Anxiety Disorder-7. *Statistically significant at p < 0.05.

Parameter	Primary school, n	Mean rank	Secondary school, n	Mean rank	University education, n	Mean rank	Kruskal-Wallis H	df	p-value
PHQ-9	8	27.81	32	35.7	20	23.25	6.515	2	0.038*
GAD-7	8	25.88	32	34.88	20	25.35	4.333	2	0.115

**Table 6:** Post hoc analysis of PHQ-9 scores by educational level among users of anxiolytics and/or sedatives. Post hoc pairwise comparisons were conducted following the Kruskal-Wallis H test, with Bonferroni correction for multiple comparisons. PHQ-9: Patient Health Questionnaire-9. *Statistically significant at p < 0.05.

Pairwise comparison	Test statistic	Standardized test statistic	Unadjusted p-value	Bonferroni-adjusted p-value
Primary school vs. university education	4.563	0.626	0.531	1
Secondary school vs. university education	12.453	2.509	0.012	0.036*
Primary school vs. secondary school	-7.891	-1.146	0.252	0.755

Among users, marital status showed no statistically significant association with the severity of depressive (H = 0.973, df = 3, p = 0.808) or anxiety symptoms (H = 0.494, df = 3, p = 0.920) (Table 7).

**Table 7:** Differences in PHQ-9 and GAD-7 scores by marital status among users of anxiolytics and/or sedatives. Differences among groups defined by marital status were evaluated using the Kruskal-Wallis H test. PHQ-9: Patient Health Questionnaire-9; GAD-7: Generalized Anxiety Disorder-7.

Parameter	Single, n	Mean rank	Married, n	Mean rank	Divorced, n	Mean rank	Widowed, n	Mean rank	Kruskal-Wallis H	df	p-value
PHQ-9	8	34.81	42	29.81	4	34.25	6	27.08	0.973	3	0.808
GAD-7	8	32.88	42	30.15	4	33.88	6	27.5	0.494	3	0.92

## Discussion

This study provides insight into the proportion of self-reported undocumented anxiolytic and/or sedative use among a single-center sample of PHC patients without a previously registered diagnosis of a mental disorder, as well as its cross-sectional association with levels of depression and anxiety. In this specific study sample, 27.1% of participants reported occasional use of anxiolytics and/or sedatives that was not recorded in their primary care medical charts at the study center. This observed prevalence reflects the characteristics of this specific clinic-based sample and should not be generalized to the broader primary care or general population of Bosnia and Herzegovina. The central finding of this research indicates that users demonstrate significantly higher cross-sectional scores for depressive symptoms (PHQ-9 median 10.0 vs. 4.0; p < 0.001) and anxiety symptoms (GAD-7 median 8.5 vs. 4.0; p < 0.001) compared with non-users. In line with the cross-sectional design, these findings reflect a statistical association rather than a causal relationship, and causality or temporal direction cannot be inferred. Furthermore, in this context, “undocumented use” refers strictly to the absence of a recorded prescription in the participant’s chart at the study center and should not be interpreted as definitive proof of non-medical or self-initiated use, as prescriptions may have been obtained through external healthcare settings.

The observed prevalence in our study (27.1%) reflects the specific characteristics of this single-center sample of PHC patients and is not directly comparable with data on non-medical use in the general populations of European countries during the preceding 12 months (ranging from 1.7% in Germany to 6.5% in Spain) [7]. This discrepancy may reflect differences in methodological approaches, such as outcome definitions and data collection methods, the absence of a standardized frequency or dose threshold in our study, and the clinic-based nature of our sample. The finding that patients reported using anxiolytics and/or sedatives without a corresponding record in their primary care charts aligns with regional observations and studies in other clinical settings [8,9]. However, because external prescribing histories and exact sources of procurement were not systematically assessed in our study, undocumented use in this sample strictly denotes the absence of a recorded prescription at the study center and should not automatically be interpreted as non-medical or self-initiated use (e.g., acquisition through informal or social networks) [11]. Variations in the observed associations between medication use and psychological burden across the literature may be explained by differences in the operational definition of use, study design, and sample characteristics [9].

It is important to emphasize that a median PHQ-9 score of 10.0 among users of anxiolytics and/or sedatives indicates moderate depressive symptom severity rather than formally diagnosed depression, as screening scales require a confirmatory clinical interview [14-16]. Given the cross-sectional design, the direction of causality cannot be definitively established: more severe symptoms may prompt self-initiated medication use, while self-reported medication use or shared underlying factors, such as stress, insomnia, and chronic pain, could also contribute to the overall burden.

In the analysis of sociodemographic factors, no significant sex- or age-related differences in symptom severity were found among users. Among non-users, however, women had significantly higher scores, which is consistent with the literature indicating a higher anxiety burden among women [17]. Although the Kruskal-Wallis H test revealed a statistically significant difference in PHQ-9 scores across age categories in the non-user group, post hoc analysis with Bonferroni correction demonstrated that this difference was restricted to the comparison between participants under 30 years of age and those aged 61-70 years, with the younger group having higher PHQ-9 rank scores. No statistically significant differences were observed among the other age groups after adjustment for multiple comparisons (all adjusted p > 0.05) (Table 4). This finding suggests that the statistically significant age-related difference in the non-user group was primarily driven by the contrast between participants under 30 years of age and those aged 61-70 years, while no significant differences were identified among the remaining age groups following post hoc adjustment.

A statistically significant difference in PHQ-9 scores by educational level was identified among users of anxiolytics and/or sedatives, with participants who had secondary education demonstrating higher PHQ-9 rank scores than those with higher education. This finding aligns with previously described associations between socioeconomic characteristics and depressive symptomatology, although the cross-sectional design and small sample sizes in certain educational categories preclude conclusions regarding causality [18].

In our sample, no statistically significant differences in the severity of depressive or anxiety symptoms were observed according to marital status, suggesting that marital status was not significantly associated with PHQ-9 and GAD-7 scores in the evaluated group. However, these results should be interpreted with caution due to the small subgroup sizes across marital status categories.

The clinical relevance of these findings highlights the need for proactive and detailed medication histories in family medicine practice. Physicians should purposefully inquire about occasional, undocumented, and self-reported use of anxiolytics and/or sedatives, particularly among patients presenting with somatic complaints, insomnia, or anxiety. Additionally, due to the number of secondary subgroup comparisons, these findings should be considered exploratory and interpreted with caution, despite the application of the Bonferroni correction for post hoc pairwise comparisons.

Methodological limitations of this study must be considered when interpreting the results. The cross-sectional design precludes establishing causal relationships between the undocumented use of anxiolytics and/or sedatives and higher depressive or anxiety scores. Furthermore, the primary comparisons between users and non-users were based on unadjusted analyses. Although key sociodemographic parameters were evaluated, potential confounding factors, such as underlying insomnia, chronic pain, somatic disease severity, or unmeasured socioeconomic stressors, were not controlled for in a multivariable model, meaning that the observed associations may be subject to residual confounding. Additionally, data on medication use were collected through self-report, introducing potential social desirability or recall bias, which may lead to an underestimation of the true prevalence of use. Because no strict numerical threshold for frequency, dose, duration, or recency was applied to define “occasional use,” participants with heterogeneous exposure patterns (ranging from rare intake to periodic use) were classified within the same user group, affecting exposure precision and direct comparability with other studies. Likewise, because prescribing history outside the study center and exact sources of procurement (e.g., private clinics, previous unrecorded prescriptions, existing home supplies, or social networks) were not captured, undocumented use should not be automatically equated with non-medical or self-initiated use. The study also did not record detailed pharmacotherapeutic parameters, such as specific drug names, exact doses, frequency, total duration of use, or time elapsed since the last dose; given that anxiolytics and sedatives represent a pharmacologically heterogeneous group, the observed associations cannot be attributed to any specific drug, dosage, or exposure pattern. An additional limitation is the relatively modest overall user group (n = 60) and the particularly small sample sizes in specific sociodemographic categories (e.g., n = 6 in the <30-year age group and n = 8 in the primary-education group). This limits statistical power and means that estimates from these small subgroups may be unstable, precluding firm conclusions. Finally, the assessment of chronic medical conditions was restricted to a binary evaluation of their presence or absence, without a more detailed assessment of specific diagnoses, clinical severity, or the number of comorbidities, which could independently influence levels of anxiety and depression.

## Conclusions

Undocumented use of anxiolytics and sedatives represents a notable phenomenon among PHC patients without a formal diagnosis of a mental disorder. Patients who report undocumented use of these medications demonstrate higher cross-sectional levels of depressive and anxiety symptoms compared with non-users. Among users, participants with secondary education exhibited significantly higher depressive scores than those with university education, whereas sex, age, and marital status showed no significant overall associations with symptom severity.

Given the cross-sectional nature of these findings and the absence of detailed pharmacotherapeutic data, these results offer a potential clinical consideration for primary care providers: maintaining awareness and considering proactive inquiry regarding unrecorded psychotropic medication use during routine consultations. Furthermore, future research in Bosnia and Herzegovina, through larger-scale and longitudinal studies, could encompass a more detailed assessment of dosing, procurement sources, and potential causal mechanisms underlying this association.
